# A humeral shaft fracture complicated with anterior shoulder dislocation in a young male treated with modified Intramedullary nailing prior to reduction: a case report

**DOI:** 10.1186/1757-1626-2-9075

**Published:** 2009-11-23

**Authors:** Konstantinos Kazakos, Stamatis Paraschou, Nikolaos G Lasanianos, Dionysios Verettas, Dimitrios N Lyras

**Affiliations:** 1 Department of Trauma and Orthopedic Surgery, Democritus University of Thrace, Dragana, B.O. 68100, Alexandroupolis, Greece

## Abstract

**Introduction:**

Anterior dislocation of the shoulder joint with an ipsilateral fracture of the humeral shaft is a rare injury which may require demanding technical skills.

**Case presentation:**

A 33 years old male sustained a work accident. Radiographs showed an anterior dislocation of the shoulder with a transverse fracture of the middle third of the humeral shaft on the same side. The dislocation proved to be irreducible in the setting of the fracture humerus. Thus, stabilization of the shaft fracture was successfully applied with an intramedullary nail and a small antirotational plate prior to the reduction. The patient recovered full function of the shoulder.

**Conclusion:**

Performing primary intramedullary nailing of the humeral shaft fracture before manipulation of the joint resulted to an excellent outcome.

## Introduction

Anterior dislocation of the shoulder joint with an ipsilateral humeral fracture is a rare injury. The first case in the modern literature was described in 1940 [1]. Since then, 22 such cases in 17 papers have been reported by other authors. We present one case of an anterior dislocation of the right shoulder with a concomitant ipsilateral humeral shaft fracture and discuss the mechanism of the injury, the problems encountered in management and the treatment alternatives.

## Case presentation

A 33-year-old Caucasian male of Greek origin sustained a work accident. He fell from a height of 2 meters and landed on his right side. On admission he was conscious and well orientated with normal vital signs. His right shoulder and arm were painful, swollen and deformed. Clinical examination revealed a closed and neurovascular intact injury that had resulted to angulation of the arm with loss of the normal contour of the shoulder.

**Figure 1:**
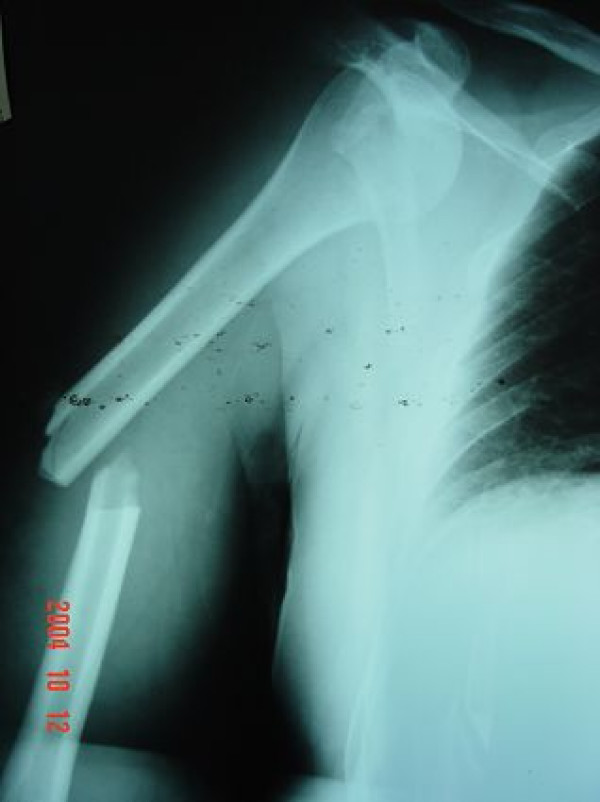
**Radiograph showing a transverse fracture of the middle-third of the right humerus with anterior dislocation of the glenohumeral joint**.

**Figure 2:**
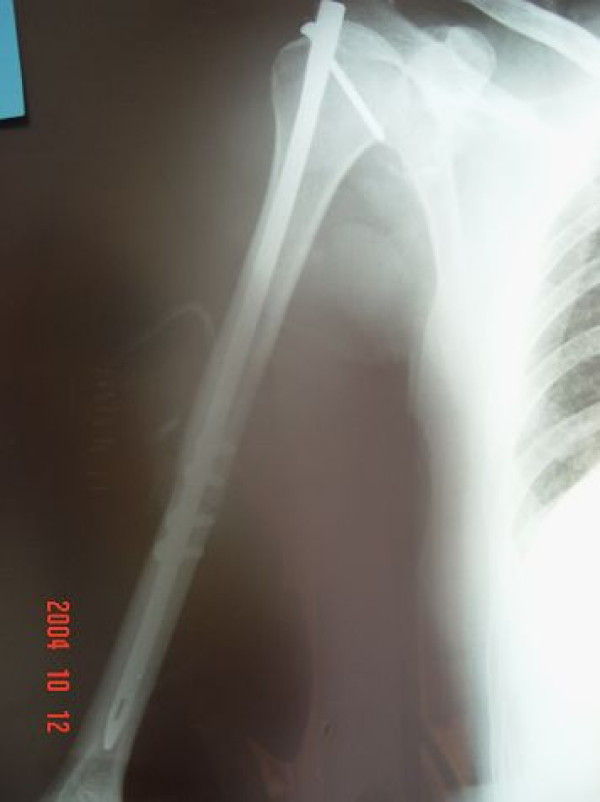
**Anteroposterior radiograph after internal fixation of the shaft fracture and reduction of the dislocation**.

Radiographs showed an anterior dislocation of the shoulder with a transverse fracture of the middle third of the humeral shaft on the same side (Figure 1). The arm was temporarily immobilized in a plaster slab. We have not performed a Magnetic Resonance scan (MRI) in order to investigate possible rotator cuff pathology, because of the lack of MRI equipment in our hospital. Thus, we decided that clinical examination at later would indicate the need or not of MRI. As soon as the secondary clinical survey was completed the patient was carried to the operating theatre. Under general anaesthesia, repeated attempts for closed reduction of the dislocated shoulder failed. Strong muscle contraction, despite the induction of muscle relaxation agents by the anesthetists, and extensive swelling of the soft tissues were obstructive of a successful reduction. Because of the unstable state of the fractured humerus and the risk for further soft tissue compromise and iatrogenic neurovascular damage, it was decided not to insist on reduction maneuvers. The surgeons preferred to attempt stabilization of the shaft fracture by intramedullary nailing prior to the reduction. Under fluoroscopy the entry point was targeted on the dislocated humeral head and an incision was made on the overlying skin. Although the anatomy of the region was altered, because of the dislocation, the anteriorly placed humeral head in the subcoracoid space facilitated, rather than obstructed, the guide wire and nail insertion. Intraoperative investigation revealed no rotator cuff or biceps pathology. Unfortunately a typical intramedullary nailing procedure could not be completed because after the proximal locking screw insertion, the image intensifier equipment suffered serious damage. The weakness for targeted distal locking which would subsequently result to rotational instability and the perspective of reduction manipulations, once the fixation was concluded, implemented the use of a rotational stability providing alternative. Thus a four holes small antirotational plate was placed on the anterior cortex over the fracture site. Four screws (2 at each side of the fracture) were inserted just medial to the nail. All screws were cortical and engaged both the anterior and posterior cortex of the bone enhancing the fixation and securing rotational stability. The shoulder was then reduced by gentle manipulation which sequentially included: external rotation, mild traction and inwards placement of the head into the glenohumeral joint (Figure 2).

**Figure 3:**
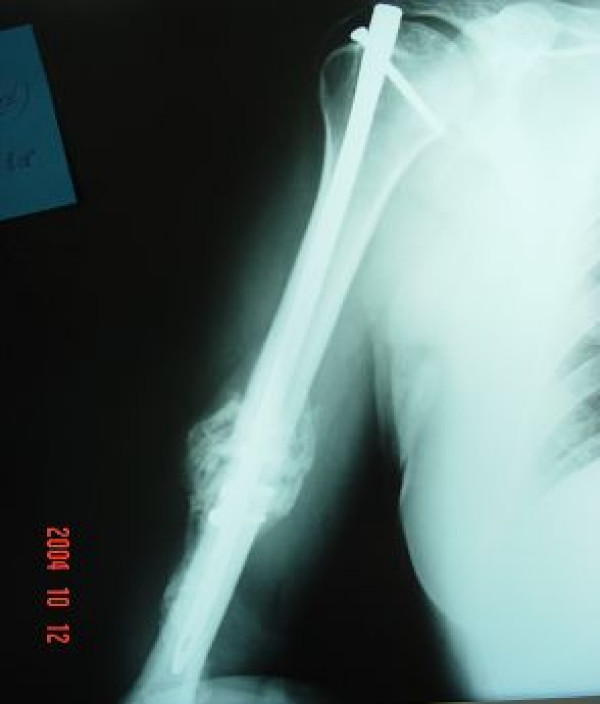
**Radiograph showing union of the fracture at 12 weeks postoperatively**.

**Figure 4:**
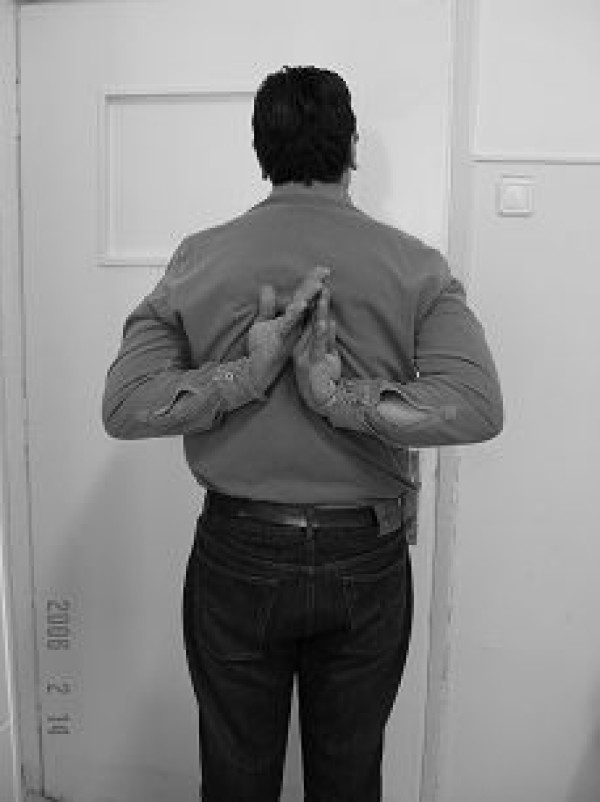
**Excellent functional results 3 years postoperatively**.

The limb was immobilized in a sling and only occasional flexion-extension of the elbow was allowed. Three weeks postoperatively the patient was encouraged to start Range of Motion (ROM) and muscle strengthening exercises of the shoulder. Bone union was demonstrated radiographically four months postoperatively (Figure 3). Mild pain and restriction of shoulder abduction over 90 degrees were demonstrated, due to the impingement of the proud nail in the subacromial space. Therefore, 12 months after the operation and since bone union was secured, both nail and plate were removed. After the hardware removal, the pain symptoms drastically subsided. Full muscular strength and normal ROM were regained. The patient returned to his daily routine activities. At 3 years follow-up, the patient's shoulder is painless and retains a full range of motion (Figure 4). No signs of shoulder osteoarthritic lesions or calcific tendonitis are evident in the latest radiographs.

## Discussion

The mechanism of the injury described is similar to that of dashboard injuries in automobile accidents where a femoral shaft fracture is associated with an ipsilateral hip dislocation [2]. The reason why the shoulder dislocates anteriorly after trauma is that as the arm extends and abducts, impingement of the greater tuberosity on the acromion levers the humeral head out of the glenoid. Moreover the rotator cuff pushes downwards the humeral head which is finally displaced anteriorly by the flexors and external rotators [3]. Our patient landed on his right side, with the elbow in flexion and the shoulder in extension and abduction. According to Sankaran-Kutty [4], in cases of combined anterior shoulder dislocation and humeral shaft fracture, the force is transmitted through the axis of the humerus to the shoulder. The energy is simultaneously distributed to the shaft of the humerus, which fractures, and to the shoulder joint. The type of injury at the shoulder depends on whether the slightly abducted arm is in some extension, flexion or in the neutral position. In the neutral position a fracture of the humeral neck or of the glenoid usually occurs. If the shoulder is extended or flexed, an anterior or posterior dislocation is produced correspondingly [4]. It is believed that the dislocation occurs first and the action of secondary forces results in the shaft fracture [5].

Various methods of treatment of this complex injury have been proposed. Closed reduction and splinting has been used in five cases with good results being achieved in 4 out of 5 occasions [1,5-7]. Closed reduction followed by external fixation has been also recommended in two reports [4,8] with satisfactory outcome. On the other hand, plate fixation has been applied in seven cases producing good results in 5 of them; however, a radial nerve palsy in one case, and a brachial plexus injury in another were recorded [5,9-11]. The use of pins has been described in two cases with good and fair outcome [12,13]. Finally, in the only report, up to our knowledge, that intramedullary nailing was used [7] good functional results were recorded.

Reviewing the relevant literature two major conclusions become evident: The first is that there is no conformity of treatment for this combined injury; the second is that in a serious number of cases [4,6,13], as in ours, closed reduction of the shoulder prior to the fracture fixation failed. The latter is more usual in cases in which the proximal fragment of the shaft of the humerus was too short to allow adequate manipulation [6]. Although closed reduction of both injuries is advocated for such complicated fracture-dislocation patterns [1,4,6,14], the risk of brachial plexus injury always exists. The use of an external fixator [4], or Steinman pins [6] as reduction tools, has been described. Moreover plate fixation prior to the reduction has been successfully used [11]. We believe though, that in the case of an irreducible anterior shoulder dislocation, combined with a humeral fracture, intramedullary nails may be more advantageous in comparison to other methods of fixation for the following reasons: (i) The insertion of the nail into the humeral head and canal is favored by the anteriorly dislocated humerus since the insertion of the nail is not obstructed by the acromion. (ii) The intramedullary positioning of the nail implant biomechanically facilitates the reduction that will follow since the risk of an iatrogenic fracture is reduced during the manipulation; in contrast extramedullary implants (plates, pins, external fixators) may create a lever capable of compromising the bone integrity. (iii) Taking into account the soft tissue swelling that usually accompanies these combined injuries, minimal invasive nailing appears preferable to large incisions demanding plating; the risk of intraoperative hemorrhage and post-operative infection is significantly reduced since the swollen soft tissues are less disturbed. (iv) Nailing is indicated for even more complicated similar fracture patterns, such as anterior shoulder dislocation with combined segmental humeral fracture; In such cases conservative treatment, plating or external fixators can scarcely produce a satisfactory result.

In our case, the treatment was determined by the circumstances. It was considered essential to attempt closed reduction of the dislocation. Since this was not possible, the next priority was to stabilize the fracture, in order to avoid at all costs open reduction of the dislocation. Considering the data provided in the previous paragraph we decided to use intramedullary nailing as a fixation procedure; however we faced two major drawbacks. The first was the damage of the image intensifier that did not allow distal locking to be applied. Since rotational stability was essential, especially in the setting of a following reduction maneuver, it was decided that a small plate should be used as an anti-rotational implant. This is not a procedure to be used as a current standard; it was necessitated though by the circumstances, and proved to be efficient. The second drawback was the improper final position of the nail. Because of wrong surgical technique, the plate that was chosen to serve only anti-rotational purposes acted as a compression plate and caused the nail to migrate proximally. Since the nail was locked proximally it would be expected that any compression on the fracture site would result to a distal rather than a proximal migration. It seems however that the loose proximal locking and the impaction of the nail in the supracondylar area, which is the narrowest part of the humeral canal [15], resulted to the opposite than the expected. Thankfully the wrongly positioned nail did not cause any permanent damage to the patient since after the hardware removal he regained a full shoulder ROM and muscle strength and his pain symptoms subsided. However his shoulder functionality was restricted during the 12 months that he carried the nail, whereas this could have been avoided with a proper technique.

## Conclusion

Several useful lessons were learned from this case: In case of an irreducible anterior shoulder dislocation combined with an ipsilateral humeral shaft fracture, primary intramedullary nailing before manipulation may provide a feasible solution with very good outcome. We would recommend it as the preferred method of fixation because it provides biomechanical advantages that facilitate the following reduction maneuvers. However, in order for the accomplishment of the nailing technique to be secured, the equipment provided should be trustworthy and there should always be a spare image intensifier available. Or else, unsafe modifications, as the one used in this case, may compromise the final result. Proximal locking should be secured with more than one screw in order for stability to be ensured. Otherwise the underlying risk of nail migration will be threatening shoulder's functionality.

## Consent

Written informed consent was obtained from the patient for publication of this case report and any accompanying images. A copy of the written consent is available for review by the Editor-in-Chief of this journal.

## Competing interests

The authors declare that they have no competing interests.

## Authors' contributions

KK and SP performed the operation, DNL and NGL prepared and revised the manuscript. DV revised the manuscript. All authors read and approved the final manuscript.
